# Supplementary material for: Disulfiram/Copper Suppresses Cancer Stem Cell Activity in Differentiated Thyroid Cancer Cells by Inhibiting BMI1 Expression
Source: Int J Mol Sci. 2022 Oct 31;23(21):13276. doi: 10.3390/ijms232113276 (PMC9654490; doi:10.3390/ijms232113276)
Supplement: Supplementary file 1 [file ijms-23-13276-s001.zip › ijms-1941089-supplementary.pdf]

Disulfiram/copper suppresses cancer stem cell activity in differentiated thyroid cancer cells by inhibiting BMI1 expression

Yung-Lun Ni[1], Peng-Ju Chien[2], Yang-Chih Hsieh[2], Huan-Ting Shen[1], Hsueh-Te Lee[3], Shih-Ming Chen[4]*, Wen-Wei Chang[2, 5]*

Supporting information

Table S1. Primer sequences used in this study

Target	Forward (5' to 3')	Reverse (5' to 3')
BMI1	AATCCCCACCTGATGTGTGT	GCTGGTCTCCAGGTAACGAA
OCT4	GGTCCGAGTGTGGTTCTGTA	CGAGGAGTACAGTGCAGTGA
SOX2	AACCCCAAGATGCACAACTC	CGGGGCCGGTATTTATAATC
NANOG	CATGAGTGTGGATCCAGCTTG	CCTGAATAAGCAGATCCATGG
MRPL19	GGGATTTGCATTCAGAGATCAG	GGAAGGGCATCTCGTAAG
BMI1-promoter (ChIP use)	CCGGGGAGAAAGAAAGAACG	CGGCCTGGGAATTAGTGTC

Table S2. Antibodies used in this study

Target	Source	Cat. No.
BMI1	Cell Signaling Technology, Inc.	6964s
c-Myc	GeneTex International Corporation	GTX109636
E2F1 (western blot)	Proteintech group Inc	12171-1-AP
E2F1 (ChIP)	BD Pharmingen	554213
Lamin B1	GeneTex International Corporation	GTX103292
GAPDH	GeneTex International Corporation	GTX100118
Tubulin	Proteintech group Inc	66031-1-Ig
HA-Tag	Santa Cruz Biotechnology, Inc	sc-7392
Mouse IgG (HRP conjugated)	GeneTex International Corporation	GTX221667-01
Rabbit IgG (HRP conjugated)	GeneTex International Corporation	GTX221666-01

Figures

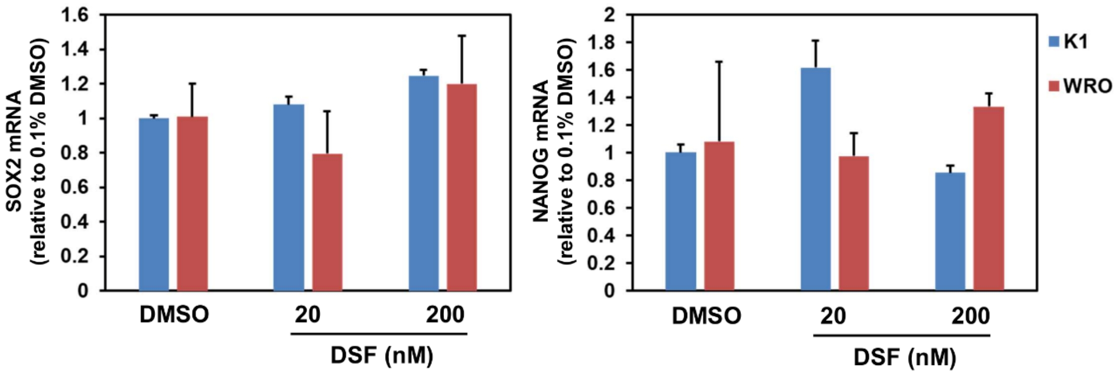

Figure S1. SOX2 or NANOG is not suppressed by DSF/copper treatment. K1 or WRO cells were treated with 20 nM or 200 nM of DSF in presence of 1 μM $CuCl2$ for 48 hours. The mRNA expressions of SOX2 or NANOG were determined by real-time RT-PCR method.

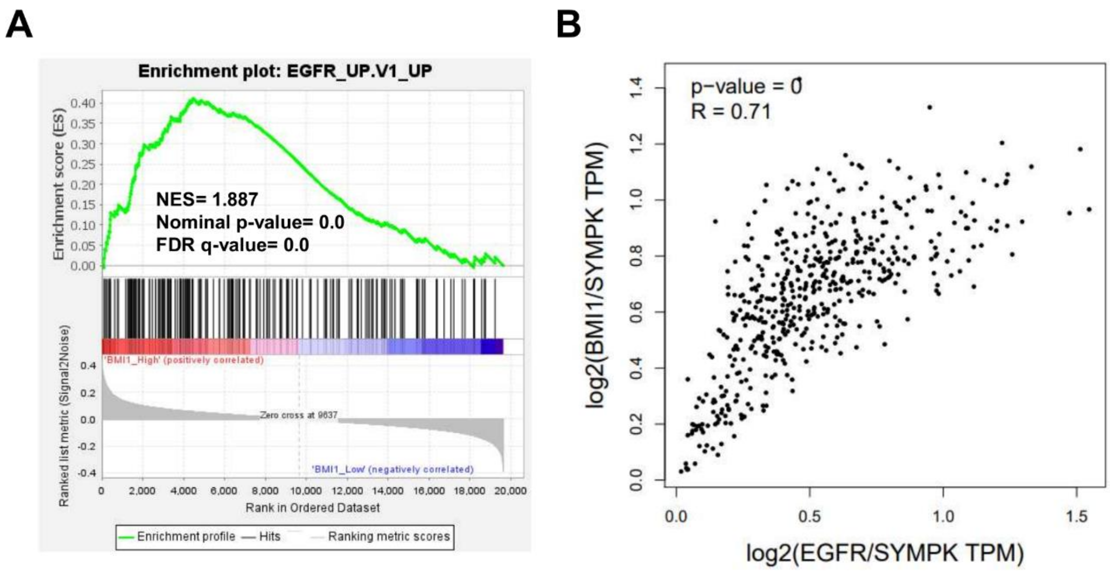

Figure S2. The positive correlation between BMI1 and EGFR among THCA samples. (A) THCA RNA-Seq dataset was obtained from TCGA database and used median expression level of BMI1 mRNA as a cutoff value followed by gene set enrichment analysis using GSEA software. BMI1 high expression samples was enriched in the gene set of EGFR_UP.V1_UP. (B) The correlation between BMI1 and EGFR in THCA dataset of TCGA data was obtained from GEPIA_2 website.

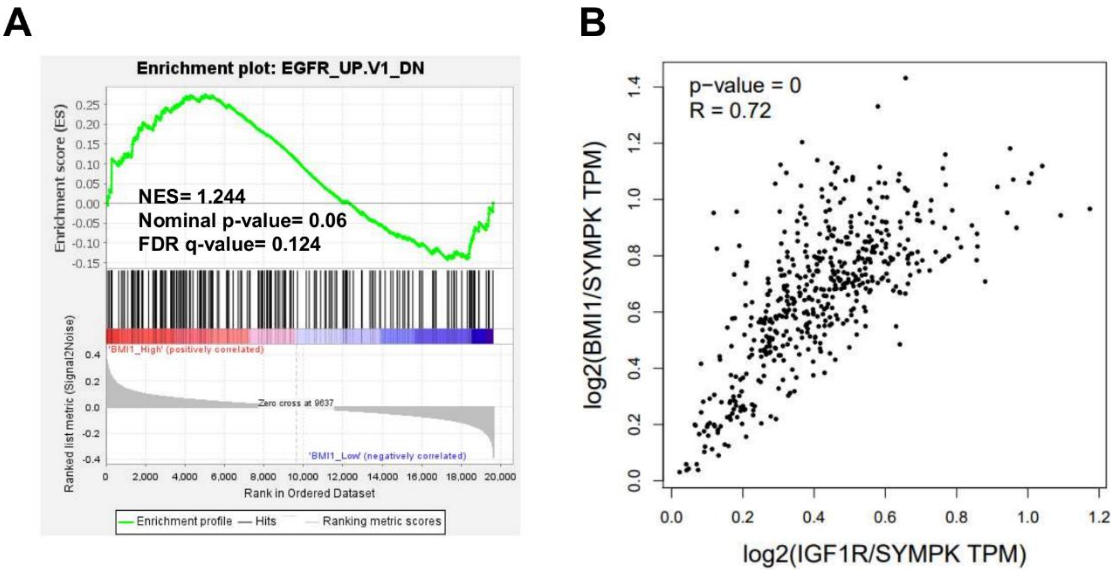

Figure S3. The positive correlation between BMI1 and IGF1R among THCA samples. (A) THCA RNA-Seq dataset was obtained from TCGA database and used median expression level of BMI1 mRNA as a cutoff value followed by gene set enrichment analysis using GSEA software. BMI1 high expression samples was enriched in the gene set of EGFR_UP.V1_ND. (B) The correlation between BMI1 and IGF1R in THCA dataset of TCGA data was obtained from GEPIA_2 website.
